# Evaluation of Poly(etheretherketone) Post’s Mechanical Strength in Comparison with Three Metal-Free Biomaterials: An In Vitro Study

**DOI:** 10.3390/polym15173583

**Published:** 2023-08-29

**Authors:** Kévin Rakotoaridina, Julien Delrieu, Paul Pages, Thierry Vergé, Karim Nasr, Thibault Canceill

**Affiliations:** 1 Département Odontologie, Faculté de Santé, Hôpitaux de Toulouse, Université Paul Sabatier, 3 Chemin des Maraichers, 31062 Toulouse Cedex 9, France; 2 CNRS UMR 5085, INPT, Faculté de Pharmacie, CIRIMAT, Université Toulouse III Paul Sabatier, 35 Chemin des Maraichers, 31062 Toulouse Cedex 9, France

**Keywords:** PEEK, glass-fiber post, post and core, prosthetic dentistry, composite materials

## Abstract

The thinking about metallic replacement has begun in a global context of reducing metallic alloys’ use in odontology. Among the materials proposed for their replacement, poly(etheretherketone) may present interesting properties, especially in removable dentures’ frames. The purpose of this study is to evaluate fracture resistance of PEEK posts-and-cores compared to non-metallic CAD/CAM materials and fiber glass posts. Forty extracted maxillary central incisors were prepared to receive posts. Samples were divided into four groups depending on whether they had been reconstructed with LuxaCam^®^ PEEK, Enamic^®^, Numerys GF^®^ or LuxaPost^®^. Samples were submitted to an oblique compressive test and results were statistically analyzed with ANOVA and Student’s tests (or non-parametric tests depending on the conditions). Glass fiber posts and Numerys GF^®^ reveal a significantly higher fracture resistance than LuxaCam^®^ PEEK and Enamic^®^. No exclusively dental fracture has been noted for the Enamic group, which significantly distinguishes these samples from the three other groups. In our study, it appears that the conception of posts and cores with hybrid ceramic never conducts to a unique tooth fracture. By weighting the results according to the materials used, our data, obtained for the first time on this type of PEEK block, cannot confirm the possibility of using PEEK for inlay-core conception, excepted for specific cases when the material is considered in a patient presenting allergies or systemic disease contraindicating resin or metal.

## 1. Introduction

There is no consensus in dentistry regarding the restoration of a severely injured tooth, with 0 or 1 healthy wall remaining on the crown after endodontic treatment. The key factor seems to be the possibility to create a ferrule effect under the crown [1]. The design of a root post is theoretically indicated in order to reinforce the tooth; however, those who oppose the use of posts consider them to be a deleterious factor [2] as a vector of intra-radicular stresses, potentiating the risk of irreversible tooth fracture.

Furthermore, the choice of the material to perform the post creates another debate because there is no consensus on this issue either [3]. Several parameters must be considered such as the need for retention (the number of posts on the same tooth and their length), the risk of material fracture and the risk of tooth fracture. In the anterior sector, the aesthetic properties of the restoration must also be taken into account [4]. It mainly exists as metal [5], fiber [6] and ceramic posts [7].

Metallic posts, also known as inlay-cores, are constituted of Cobalt–Chromium (CoCr) alloys or more rarely of Nickel–Chromium (NiCr) or Titanium alloys because of a high elasticity modulus [8]. This property allows one to increase the fracture resistance of the material, but the differences between the elasticity modulus of the post and those of the tooth may complicate the stress transmission inside the root during chewing cycles [9]. The residual tissues are thus weakened and may break [3].

The solution of using fiber posts, whose elasticity modulus is closer to the dentin [10], would improve the stress repartition inside the root [11]. They are constituted of a prefabricated post, made of a resin matrix reinforced with longitudinal fibers (of glass, carbon or quartz) and bonded to the intra-radicular dentin [12]. A strict protocol is required for their successful implementation since they imply the execution of a bonding procedure. However, with the resistance of the biomaterial being lower, more important is the risk of post fracture [13]. The main cause of failure of this approach is the dislodgment, but, with the tooth being less exposed to a fracture, its life expectancy on the arch is potentially increased [14].

A meta-analysis has recently shown that both metallic and fiber posts could be considered as valuable therapeutic solutions; the choice being driven by the practitioner’s preferences [7]. The thinking about metallic replacement has begun in a global context of reducing metallic alloys’ use in odontology, especially because of the evolution of the regulatory frameworks all over the world concerning these metals’ security of use [15].

The progress of digital dentistry and CAD/CAM devices has been accompanied by the development of new materials, available as blocks or discs and shaped by machining after computer design of the piece to be manufactured. A block supposed to reproduce a fiber-reinforced post (Numerys GF, Itena Clinical, France), for example, was launched in 2019. According to the manufacturer’s data, it is composed of 20% to 25% of epoxy resin and 75% to 80% of unidirectionally oriented glass fibers, and it presents a modulus of elasticity of approximatively 25 GPa. Earlier, in 2013, there have appeared the new Polymer-Infiltrated Ceramic Networks (PICN) composed of 86% ceramic and 14% polymers for the first generation (Enamic, Vita Zahnfabrik, Germany). Mainly intended for use in coronal reconstruction, their modulus of elasticity, close to 30 GPa, has led to imagining their use as root posts [16,17].

Poly(EtherEtherKetone) (PEEK) is a semi-crystalline polyaromatic thermoplastic polymer which has been marketed in the industry since the early 1980s [18]. Its first biomedical application was developed in the late 1990s [19] as an alternative to metallic or ceramic medical devices in orthopedics or craniofacial reconstructive surgery. It began to be used in the field of odontology more than 10 years ago to create removable partial denture’s basis, several elements in implantology (fixtures abutments, healing screws) [20] or even occlusal splints [21]. This material is free of corrosion and radiolucent [22,23] which offers an interesting comparison with metals. It is biocompatible, non-toxic, stable over time [24] and hypo-allergenic in the absence of monomers in its structure [25,26]. Even if the applications for PEEK in dentistry are numerous in prosthodontics [20], only a few publications have already studied the potential for this biomaterial to serve for post conception.

The objective of our study will thus be to evaluate the mechanical strength of PEEK posts in comparison with three other non-metallic biomaterials. The null hypothesis is that the type of material does not influence the fracture resistance of the tooth-restoration assembly.

## 2. Materials and Methods

An in vitro study has been performed in Toulouse Health Faculty (Département Odontologie, Université Toulouse III, Toulouse, France).

### 2.1. Teeth Collection

Forty human upper incisors, extracted for periodontal reasons, have been collected in Toulouse Hospital (Service d’Odontologie, Hôpitaux de Toulouse, Toulouse, France) in compliance with the Hospital’s rules for tissue collection. Before the beginning of the study, the teeth were conserved in a 1% chloramine solution in order to decontaminate them as well as maintain their hydration and their integrity before mechanical tests [27]. To be suitable for use in the study, the teeth had to present healthy crown and root—i.e., those who present a decay, a crack, a fracture, an incomplete root formation, a resorption, endodontic calcification (evaluated on an X-ray) or a root curvature higher than 20° have been excluded.

### 2.2. Teeth Preparation

**Figure 1:**
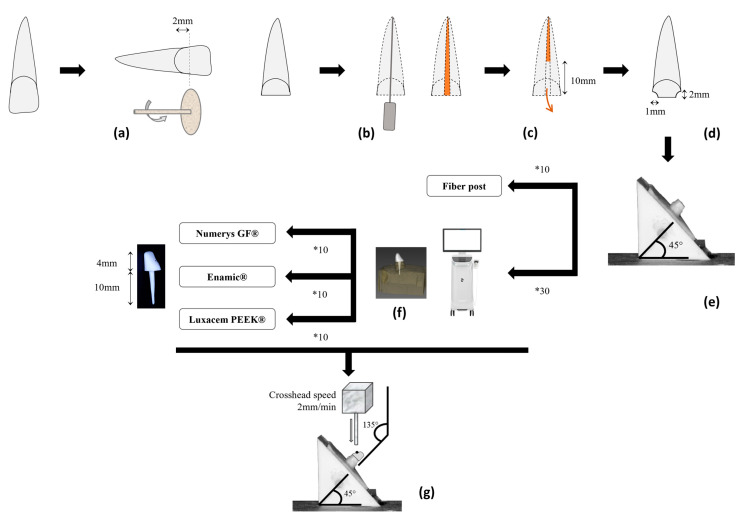
Schematic representation of the different steps of the experimental procedure. Briefly, each incisor’s crown was cut (**a**) and the tooth treated endodontically (**b**). Next, 10 mm of the endodontic filling was removed (**c**), a corono-peripheral preparation was performed (**d**) and each tooth was embedded in standardized resin mold (**e**). Among the 40 teeth, 10 received a fiber post, and the 30 others were scanned with an intra-oral scanner to design and produce posts and cores (**f**) (10 in fiber-reinforced resin, 10 in hybrid ceramic, 10 in PEEK). Once all the restorations were bonded to the endodontic dentin, the samples were submitted to a universal testing machine in a compression mode at a crosshead speed of 2 mm/min (**g**).

The crowns of the teeth were sectioned perpendicularly to their longitudinal axis, 2 mm above the buccal cement-enamel junction, with a low-speed diamond disc under irrigation (IsoMet 2000, Buehler, Evanston III, Leinfelden-Echterdingen, Germany) (Figure 1a). This cut allowed us to access the endodontic canal whose preparation was performed with a constant rotation system (ProTaper Next, Dentsply-Sirona, Bensheim, Germany). The X2 final file had a 025 diameter and a 6% conicity. Each instrument passage was followed by a 3% sodium hypochlorite rinse, completed for final irrigation with a 17% ethylenediaminetetraacetic acid (EDTA) rinse. The endodontic obturation (Figure 1b) had been performed following the thermocompacted monocone technique with an adequate gutta-percha cone and a eugenol-free cement (AH Plus, Dentsply-Sirola, Bensheim, Germany) to anticipate the need for bonding procedures with the future posts. Then, the endodontic treatment was removed for the first 10 mm of the canal (Gates #3, Dentsply-Sirona, Germany) (Figure 1c) and the endodontium was flared with the smaller file of the fiber post kit (LuxaPost, DMG, Hambourg, Germany). The endodontic preparation ended with a final rinse and drying. A 1 mm wide corono-peripheral reduction was made for each tooth, defining a 2 mm high dentinal cerclage (Figure 1d) in accordance with the minimum ferrule height of 1.5 to 2 mm recommended in the literature [28,29].

The prepared roots have been included in a self-curing acrylic resin (SR Ivolen, Ivoclar Vivadent, Saint-Jorioz, France) inside a pyramid-shaped mold that produces a 135° angle between the probe of the future fracture test and the tooth (Figure 1e). This angle simulates the natural inter-incisal angle formed by the longitudinal axes of the maxillary and mandibular central incisors and has already been reproduced many times in mechanical studies [30,31,32,33,34,35,36,37,38].

### 2.3. Samples Design and Assembly

The 40 prepared teeth were randomly assigned to one of the four groups (*n* = 10), characterized individually by a different biomaterial to conceive the post: the LuxaCam PEEK (DMG, Hambourg, Germany), the Enamic (Vita Zahnfabrik, Bad Säckingen, Germany), the Numerys GF (Itena Clinical, Villepinte, France) and the LuxaTemp fiber post (DMG, Hambourg, Germany). Their main characteristics are summarized in Table 1.

For the control group (fiber post LuxaPost small, DMG, Hambourg, Germany), the intra-root canal preparations were etched with 35% orthophosphoric acid (Vococid, VOCO, Cuxhaven, Germany) for 20 s and then rinsed and dried again. A universal adhesive (PermaBond Universel, DMG, Hambourg, Germany) was applied inside the preparations and dried before a 20 s light-curing sequence. The fiber post contained in the kit is already coated with a silane, so the adhesive was directly applied on the post, dried and light-cured for 20 s. A dual-cure restorative composite resin (LuxaCore, DMG, Hambourg, Germany) was injected into the endodontic canals before the fiber post insertion. A 40 s photopolymerization was performed. Then the same composite resin was applied again to build-up the core, and a final light-curing sequence of 20 s was launched. According to the manufacturers’ recommendations, the biomaterial’s curing is complete 5 min after the end of this whole protocol. The design of the occlusal core was thought to measure 4 mm high, the same dimension as the machined posts in the other groups.

For the three other groups, the endodontic preparations have been registered with an intra-oral scanner (Cerec Primescan, Dentsply-Sirona, Bensheim, Germany) whose depth of field of 20 mm was able to access the more apical part of the cavity. The various posts and cores had been designed on Inlab software (Inlab 18.1, Dentsply-Sirona, Bensheim, Germany) before launching the drilling machine (MCXL, Dentsply-Sirona, Bensheim, Germany) with the three types of CAD/CAM blocks (Figure 1f).

The inlay-cores made of PEEK-based material have been sandblasted (50 µm Alumine, 2 bar) and air-dried. A coupling agent (LuxaTemp Glaze & Bond, DMG, Hambourg, Germany) was then slightly applied and light-cured for 20 s. For the hybrid-ceramics restorations, a 5% fluorhydric acid gel (Adiva Cera Etch, Vita-Zahnfabrik, Bad Säckingen, Germany) was applied for 60 s; then the pieces were rinsed, and a silane (Adive C-Prime, Vita-Zahnfabrik, Bad Säckingen, Germany) was coated on their surface. The inlay-cores made of Numerys GF were coated with a specific silane (Silane-it, Itena Clinical, Villepinte, France) only. All these restorations have been bonded on dental tissues with a self-adhesive resin (Permacem 2.0, DMG, Hambourg, Germany) following a principle of double application: one on the post and one inside the tooth. Once the posts were inserted into the roots, the resin excesses were cleaned with a microbrush during the chemopolymerization’s waiting period (30 s under digital pressure and 7 min with no pressure). The final step of the protocol consisted of light-curing buccal and lingual faces of the teeth

### 2.4. Fracture Tests

The samples, included in their acrylic bases, were submitted to a constant oblique compressive stress at a crosshead speed of 2 mm/min (TA.XT Plus Texture Analyzer, Stable MicroSystems, Godalming, UK) (Figure 1g). The point of impact of the load was located at 2 mm from the incisal edge, in the middle of the lingual surface. The cylindrical probe was positioned to form a 135° angle with the longitudinal axis of the tooth (Figure 1g). Fracture was defined as the point at which the stress reached its maximum value by fracturing the material, the tooth or both. The strength and tenacity values were calculated.

### 2.5. Fracture Mode

The fracture mode of each tooth has been analyzed after the mechanical tests and classified into one of the three possibilities: dental (only the tooth was broken, the post being intact), material (only the post was broken, the tooth-integrity being respected) or mixed (both the tooth and the post were broken).

### 2.6. Statistical Analyses

All the data have been collected on an Excel sheet (Microsoft Excel 2019, Microsoft, Redmond, WA, USA). Then the analyses and figures were designed on Stata (Stata v.13, StataCorp, College Station, TX, USA) and Prism (Prism 5, GraphPad, Boston, MA, USA) software. Comparisons between the groups have been performed with ANOVA (global *p*-value) or Student’s tests (bivariate analyses) after verification of the values’ normal distribution and variance equality. If Shapiro–Wilk tests (for normality) and/or Levene’s tests (for variance equality) were significant, non-parametric Mann–Whitney tests were preferred for bivariate analyses. The fracture mode being presented as a classification, Fisher tests have been used to compare groups. The significance level was fixed at 5%.

## 3. Results

### 3.1. Fracture Tests

Posts made of PEEK-based biomaterial present a lower resistance (9.48 ± 6.65 MPa) than fiber posts (15.88 ± 4.37 MPa, *p* = 0.005) and Numerys GF posts (15.35 ± 6.65 MPa, *p* = 0.03) (Table 2). The Enamic biomaterial is the option for which the lower strength is necessary to reach the fracture (6.05 ± 4.14 MPa; *p* = 0.0001 and 0.002 in comparison respectively with fiber posts and Numerys GF posts).

Tenacity, that can be interpreted as the energy stored until failure, is highest for the fiber posts (12.58 ± 5.46 Pa/m²), and these are significantly more elevated than those of PEEK (6.43 ± 4.22 Pa/m², *p* = 0.01) and Enamic posts (3.18 ± 2.92 Pa/m², *p* = 0.0005). For the Numerys GF posts, tenacity is only significantly different than those of the Enamic group (9.69 ± 5.92 Pa/m², *p* = 0.002).

### 3.2. Fracture Mode

**Figure 2:**
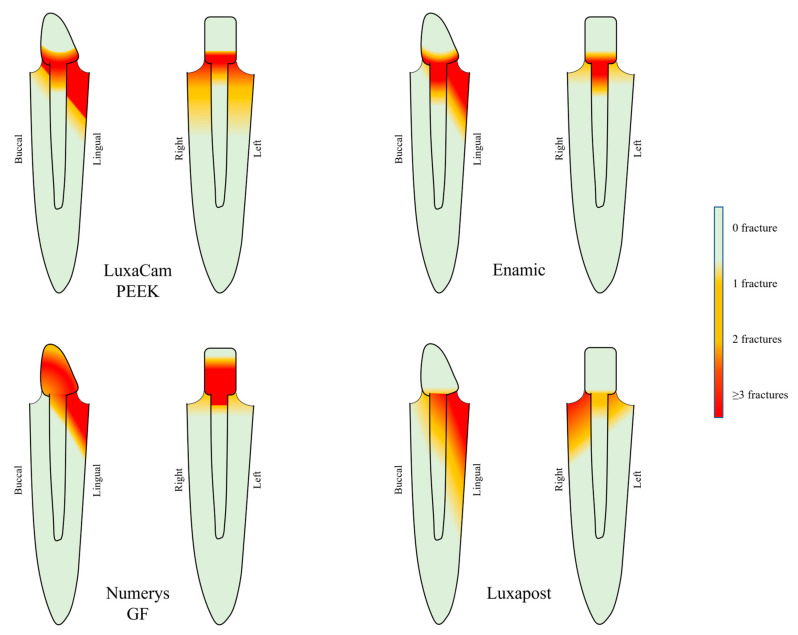
Distribution of the fracture lines retrieved among the samples of each of the four groups. For each material, side (left) and front (right) diagrams are shown. Each group brings 10 samples.

No exclusively dental fracture was noted for the Enamic group (Figure 2), which significantly distinguishes the proportions of fracture patterns of these samples from the three other groups (60% of dental fracture in PEEK group (*p* = 0.02), 80% in fiber posts group (*p* = 0.001) and 70% in Numerys GF group (*p* = 0.001)). All the modes of fracture are summarized in Table 3.

As shown in Figure 2, the fracture feature profiles on the material are mainly located at the post-core junction for the PEEK and Enamic samples. In the Numerys GF^®^ group, fractures are more widespread on the core.

## 4. Discussion

The results of our study highlight the excellent mechanical behavior of fiber-reinforced materials (fiber posts and Numerys GF) in comparison with PEEK-based material and hybrid ceramic for the conception of post and core reconstitutions. The null hypothesis initially formulated concerning the absence of influence of the material type on the fracture resistance is rejected.

These results are consistent with the real indications for each one of these materials. PEEK and hybrid ceramic are sold for a use, respectively, in removable dentures and fixed prosthesis (inlays and crowns). The key element to explain the values obtained is the biomaterials’ elasticity modulus because the results are better for the LuxaPost and the Numerys GF, which are the two materials for which the Young modulus is close to those of the dentin [40]. Dietschi et al. have concluded, in their systematic review more than 15 years ago, that the strength experienced by a tooth would be better distributed along the root with a material whose elasticity modulus is closer to natural tissues [8].

In our study, it appears that the conception of posts and cores with hybrid ceramic never conducts to the tooth fracture. This characteristic makes one question the possibility of using this material in this way. On the one hand, it is difficult to consider a restoration with a very low tenacity when milled as a post. On the other hand, its poor mechanical resistance may be interesting for the root survival, as a biomaterial that breaks with no injury on dental tissues would improve the “life expectancy” of the tooth and make the new therapeutic intervention easier. Our results must be balanced with regard to the existing literature, especially previously published results that have shown the absence of a significant difference in the fracture resistance of hybrid ceramic, zirconium and metallic inlay-cores [16]. However, the authors have also noted fewer root fractures in the hybrid ceramic group [16].

An important number of studies have already compared the in vitro and clinical behaviors of fiber posts and metallic posts [13,14,41,42,43,44,45,46,47,48]. Among them, two randomized clinical trials have concluded in the absence of significant difference between the two types of tenons [41,42]. Their survival after a follow-up period of 17 years is comparable [43]. Our study was focused on upper incisors with aesthetic considerations and with a metal-free thinking; that is why no metallic inlay-core was included here, even if they present an excellent fracture resistance [13].

The material that is still relatively new and, therefore, lacks such clinical feedback is the milled fiber-reinforced post (Numerys GF). In contrast with the conventional fiber post, it is especially adapted to the dentin walls; thus, the adhesive thickness is lower, and the risks for air bubbles and gaps are reduced [17]. Our results, which show the similar resistance of the two types of restorations, confirm the existing data obtained by Eid et al. on a standardized fiber post and a milled one [49]. However, we have chosen to concentrate purely on mechanical behavior with no intervention of fatigue in our tests, on which others have conducted research, such as Falcão Spina et al. in 2017 [17]. Their results slightly differ since they showed no difference between several milled esthetic biomaterials used as posts including hybrid ceramic [17]. The greatest proximity of values in their observations may be explained by the lower tolerance of fiber-reinforced resins to fatigue in comparison with those of Enamic to fatigue, which probably smoothed their results in contrast to those we have obtained. The interest of fatigue simulation is to apply a dynamic repeated stress that reproduces the real occlusal strength in humans and, thus, to accelerate the material wear [8]. It is interesting to highlight that one study, published in 2019, shows an important fracture resistance for hybrid ceramic posts and cores (793.8 ± 55.6 N in Enamic group vs. 607.7 ± 54.8 N in fiber posts group) on samples that were submitted to thermocycling aging and entirely restored since the authors had added metallic crowns on the cores [50]. The presence of a crown obviously improves the clinical relevance of the mechanical test, but its shape, its thickness and its composition add a new resistance to the whole restoration and may hide the specific properties of the post [51].

Previously published works on PEEK used for post design are relatively recent even if the biomaterial has been of interest in dentistry for approximatively two decades. Thanks to new technologies, Sammany et al. in 2019 [40], Yu et al. in 2022 [52] and Gontijo et al. more recently [53] have performed finite elements tri-dimensional analyses to evaluate the behavior of PEEK inlay-cores, and their studies have shown that the tooth survival was not compromised when it was reconstituted with such a post [40,53]. Interestingly, the results of Sammany et al. show that under physiological forces, the stress distribution all along the root was similar to those of a safe tooth [40]. In 2020, Teixeira et al. have compared the fracture resistance of several biomaterials designed as posts and cores with interesting results concerning Poly(EtherEtherKetone) [54]. Indeed, they found no significant difference between this material, fiber posts and milled composite resin. Among the failures observed in the PEEK group, 83.3% were a loss of bonding and could thus be considered as favorable for tooth survival [54]. Recently, in 2023, M.O. Lima et al. have published results showing that PEEK posts and cores were slightly similar to those of glass-fiber posts in tooth resistance preservation provided that a ferrule effect is respected during tissue preparation [55]. The main characteristics of the protocols already published that have evaluated the resistance and feasibility of PEEK posts and cores are presented in Table 4.

Some of our results may be contradictory in comparison with others studies, especially one published this year. The study used other types of PEEK CAD/CAM blocks and showed material deformation only for PEEK whereas posts made of Enamic^®^ critically broke in 20% of the cases [56]. As presented in Table 4, the PEEK CAD/CAM blocks used in our study have never been tested previously. Thus, our results, that highlight that PEEK posts and cores seem less resistant than fiber-reinforced posts (pre-fabricated or milled) and that 80% present tooth fracture, should be weighted by limiting their external validity to these blocks in particular. Moreover, our protocol slightly differs with those retrieved in previous publications, as we chose to perform a tissue preparation before sealing the posts in order to create a ferule effect on the tooth. This parameter was also designed in only 5 publications of the 22 published since 2020 [53,55,57,58,59]. It may also be worth highlighting one of the parameters that varies greatly between the different studies, namely the type of support on which the tests have been carried out. Without taking into account numerical simulation analyses, in vitro tests have been carried out on natural human teeth [56,59,60], bovine teeth [55,57,61] or artificial supports [62,63], which can significantly change the results obtained. Finally, the last parameters that may influence the differences observed between the studies concern the nature of the assembling biomaterial or the post’s size. Here, a self-adhesive resin containing mainly metacrylic esters (Permacem 2.0, DMG, Hambourg, Germany) has been chosen for three reasons: its compatibility with the three types of milled posts, its easy-handling and, thus, the reduction of bias in its manipulation between several samples. In the majority of studies, a self-adhesive resin with tri-ethylene glycol dimethacrylate (TEGDMA) monomer is used (RelyX Unicem, 3 M ESPE, St Paul, MN, USA) [17,49,54,64,65,66]. The height of the tenons is directly correlated to the thickness of the residual tissues, whatever the type of post, the health of these tissues, or the therapeutic’s success rate [8,13,14,44,45]. Over the root, the presence of a ferrule effect may be decisive in the prosthetic survival [1,8,67,68,69,70]. It is efficient when there is a dentin wall perpendicular to the cervical limit on the whole circumference of the tooth. Its objective is to reduce the intra-canal stress and consequently increase its resistance to fracture [67]. There are conflicting data in the literature concerning its exact dimensions, but it seems to be recognized that the ferrule effect should have a minimal size of 1 mm height [8,70], a 0.5 mm thickness [68] and a presence on at least 75% of the tooth’s circumference [1]. According to Naumann et al., the presence of the ferrule is more important than the choice of the post [1]. Pascal Magne et al. have even suggested that a tenon would no longer be necessary if the ferrule were higher than 2 mm in height and 1 mm in thickness [69]. If a post is chosen, its length should not be considered a decisive factor in the bonding protocol applied [71] as the number of dentinal tubules decreases from the coronal to the apical part of the root. Apically, the tissues are less accessible to instrumentation and photopolymerization light [71], so it appears not to be necessary to increase the tenon dimensions just to improve the quality of the bonding procedure.

Our data thus cannot confirm the possibility of using PEEK for inlay-core conception, but it is possible to mention specific cases in which it can be of high interest when the material is considered in a patient presenting allergies or systemic disease contraindicating resin or metal [21].

**Table 4:** Experimental conditions applied in the studies published since 2020 in which the authors have evaluated the mechanical properties of PEEK posts and cores.

Year of Pub.	Authors	PEEK Manufacturer	Main Characteristics of the Protocol	Main Conclusions on PEEK
2023	Our study	DMG	Oblique compressive stress (2 mm/min) on PEEK posts and cores bonded to natural teeth (with tissue preparation and ferule effect).	Resistance of PEEK is lower than those of fiber posts.
2023	Ahmad et al. [59]	Juvora	Oblique compressive stress (1 mm/min) on PEEK posts and cores bonded to natural teeth (with tissue preparation and ferule effect) and pull-out tests.	Higher resistance for prefabricated PEEK posts with composite core than PEEK full posts and cores. Similar results for pull-out tests.
2023	Gontijo et al. [57]	Juvora	Oblique compressive stress (0.5 mm/min) on PEEK posts and cores bonded to bovine teeth (with tissue preparation and ferule effect). Moreover, simulation with finite element analysis.	Weakened roots restored with PEEK posts and cores are more resistant than those treated with prefabricated glass fiber posts.
2023	Kole et al. [60]	Juvora	Push-out stress on slices of PEEK posts and cores bonded to natural teeth (no ferule effect).	Zirconia post-cores appear to be a promising material.
2023	Lima et al. [55]	Juvora	Oblique compressive stress (1 mm/min) on PEEK posts and cores bonded to bovine teeth (with tissue preparation and ferule effect). Moreover, simulation with finite element analysis.	Fracture resistance of the tooth is not different with PEEK posts and cores.
2023	Saisho et al. [56]	Amann Girrbach	Oblique compressive stress (1 mm/min) on PEEK posts and cores bonded to natural teeth (no ferule effect) and pull-out tests.	No difference between the materials for compressive load. PEEK posts and cores showed lower bond strength to intracanal dentin.
2023	Zhao et al. [62]	Unspecified	Shear bond strength of PEEK-glass fibers composites cores on artificial molds. No posts; no use of natural teeth.	The mechanical properties of the composites were greatly improved.
2022	Attia et al. [72]	Bredent	Pull-out tests on PEEK posts and cores bonded to natural teeth (no ferule effect).	Precisions on bonding protocol, not on material choice for post and core (no control group).
2022	Gontijo et al. [53]	Juvora	Oblique compressive stress (0.5 mm/min) on PEEK posts and cores bonded to bovine teeth (with tissue preparation and ferule effect).	In presence of good bone condition, roots restored with PEEK posts and cores provide more reparable fractures and more resistant roots than those treated with prefabricated and anatomic glass fiber posts.
2022	Hallak et al. [73]	Informatic simulation	Simulation of masticatory forces with finite element analysis software on upper central incisors restored with PEEK posts.	Similar stress intensity and distribution between PEEK and glass fiber posts.
2022	Monteiro et al. [61]	Bredent	Pull-out tests on PEEK posts and cores bonded to bovine teeth (no ferule effect).	Good clinical options, but with a need for improvement.
2022	Pourkhalili et al. [74]	Bredent	Oblique compressive stress (0.5 mm/min) on PEEK posts and cores bonded to natural teeth (ferule effect not specified).	Mode of failure mostly repairable in the PEEK group. Resistance is lower than Ni-Cr alloys and greater than fiberglass posts.
2022	Yu et al. [52]	Informatic simulation	Simulation of mechanical load with finite element analysis software on upper central incisors restored with PEEK and Carbone-reinforced-PEEK posts.	Biomechanical behavior of the CFR-PEEK posts and cores was the closest to dentin.
2021	Haralur SB. [75]	Unspecified	Oblique compressive stress (0.5 mm/min) on PEEK posts and cores bonded to natural teeth (with tissue preparation).	Resistance of PEEK is higher than those of fiber posts.
2021	Ibrahim et al. [76]	Informatic simulation	Simulation of mechanical and thermal load with finite element analysis software on upper central incisors restored with PEEK posts.	Good resistance to masticatory forces for teeth restored with PEEK posts and favorable intra-radicular stress distribution.
2021	Özarslan et al. [77]	Juvora	Oblique compressive stress (1 mm/min) on PEEK posts and cores bonded to natural teeth (no ferule effect).	No superior features for PEEK in comparison with zirconia and glass-fiber.
2020	Benli et al. [78]	Juvora	Pull-out tests on PEEK posts bonded on natural teeth. No cores.	PEEK is a reliable option for dental posts.
2020	Çulhaoglu et al. [63]	Bredent	Shear bond strength of PEEK cores on resin. No posts; no use of natural teeth.	Acceptable resistance of resin bonding on PEEK surface.
2020	Li et al. [79]	Bredent	Pull-out tests of PEEK post and core restorations combined with polyvinylsiloxane attachments. No use of natural teeth.	The new post and core system showed favorable retention forces.
2020	Nahar et al. [80]	Informatic simulation	Simulation of mechanical load with finite element analysis software on upper central incisors restored with several PEEK posts (carbon fibers-reinforced (CFR), glass fibers-reinforced (GFR), PEKK).	CFR-PEEK is a good material for the fabrication of endodontic post. GFR-PEEK and PEKK materials can also be used.
2020	Sugano et al. [58]	Yasojima	Oblique compressive stress (1 mm/min) on PEEK posts and resin cores bonded to bovine teeth with flared canals (with ferule effect).	Higher stability is obtained with PEEK posts and glass fiber sleeves.
2020	Teixeira et al. [54]	DEGOS	Oblique compressive stress (0.5 mm/min) on PEEK posts and cores bonded to natural teeth (no ferule effect).	Resistance of PEEK is comparable to those of fiber posts.
2020	Tekin et al. [81]	Informatic simulation	Simulation of mechanical load and stress distribution with finite element analysis software on upper central incisors restored with PEEK posts and cores.	No difference in the stress value between PEEK and fiber posts.

## 5. Conclusions

This study has highlighted the fracture resistance of several biomaterials used to build-up bonded posts and cores on natural human teeth. Within the limitations of this study, the following conclusions could be drawn:

1. Under oblique compressive tests, posts and cores designed from CAD/CAM blocks made of PEEK (80%) and TiO_2_ (20%) present a lower resistance in comparison to fiber posts.
2. Prefabricated fiber posts and milled fiber posts present a similar behavior under oblique compressive tests.
3. The Enamic material, used to design posts and cores, seems to be the most protective concerning root fracture.

The results of this study need to be weighted according to block manufacturers, particularly in the case of PEEK.

## Figures and Tables

**Table 1:** Main characteristics of the four biomaterials used to conceive the posts in this study (data extracted from [39] and manufacturers’ brochures).

Biomaterials	Manufacturer	Composition	Elasticity Modulus (GPa)
LuxaCam PEEK	DMG	PEEK 80%; TiO_2_ 20%	3.8
Enamic	Vita	Ceramic 84%; Polymers 14%	30
Numerys GF	Itena	Glass fibers 75–80%; Epoxy resin 20–25%	25
LuxaPost	DMG	Glass fibers; bis-GMA resin	25

**Table 2:** Results of the fracture tests performed on the four materials. Values are presented as Mean ± Standard Deviation. A different letter ^a,b,c^ in the same line indicates that the difference between the two biomaterials presents a *p*-value lower than 0.05. Each group contains 10 samples.

	LuxaCam PEEK	LuxaPost	Numerys GF	Enamic
Maximal strength (MPa)	9.48 ± 6.65 ^a^	15.88 ± 4.37 ^b^	15.35 ± 6.65 ^b^	6.05 ± 4.14 ^a^
Tenacity (Pa/m²)	6.43 ± 4.22 ^a,c^	12.58 ± 5.46 ^b^	9.69 ± 5.92 ^b,c^	3.18 ± 2.92 ^a^

**Table 3:** Proportion of each fracture mode observed in the four groups. A different letter ^a,b,c,d^ indicates that the difference between the two biomaterials presents a *p*-value lower than 0.05. Each group contains 10 samples.

Fracture Mode	LuxaCam PEEK ^a,b^	Luxapost ^b,c^	Numerys GF ^a,c^	Enamic ^d^
Dental	80%	60%	70%	0
Material	10%	20%	0	60%
Mixed	10%	20%	30%	40%

## Data Availability

Data are available on reasonable demand addressed to the authors.
